# The role of systemic immuno-inflammatory factors in resectable pancreatic adenocarcinoma: a cohort retrospective study

**DOI:** 10.1186/s12957-022-02606-1

**Published:** 2022-05-06

**Authors:** D. Schlanger, C. Popa, S. Pașca, A. Seicean, N. Al Hajjar

**Affiliations:** 1 “Iuliu Haţieganu” University of Medicine and Pharmacy, Cluj-Napoca, Romania. Street Emil Isac no. 13, 400023 Cluj-Napoca, Romania; 2 Surgery Department, Regional Institute of Gastroenterology and Hepatology “Prof. Dr. O. Fodor”, Cluj-Napoca, Romania. Street Croitorilor no. 19-21, 400162 Cluj-Napoca, Romania; 3 Department of Haematology, “Iuliu Haţieganu” University of Medicine and Pharmacy, Cluj-Napoca, Romania. Street Emil Isac no. 13, 400012 Cluj-Napoca, Romania; 4 Gastroenterology Department, Regional Institute of Gastroenterology and Hepatology “Prof. Dr. O. Fodor”, Cluj-Napoca, Romania. Street Croitorilor no 19-21, 400162 Cluj-Napoca, Romania

**Keywords:** Pancreatic cancer, Systemic inflammatory response, Cancer prognosis, Prognostic nutritional index, Platelet to lymphocyte ratio

## Abstract

**Background:**

Pancreatic cancer is an aggressive malignancy, surgery being the only potentially curative treatment. The systemic inflammatory response is an important factor in the development of cancer. There is still controversy regarding its role in pancreatic cancer.

**Methods:**

Our study is a retrospective observational cohort study. We included patients diagnosed with pancreatic ductal adenocarcinoma (PDAC), who underwent surgical resection in our hospital, between January 2012 and December 2019. We gathered information from preoperative and postoperative blood tests. Neutrophil to lymphocyte ratio (NLR), platelet to lymphocyte ratio (PLR), lymphocyte to monocyte ratio (LMR), prognostic nutritional index (PNI) and systemic immune-inflammation index (SII) were determined.

**Results:**

We included 312 patients. All the immune-inflammatory scores assessed significantly changed after the surgery. The impact on overall survival of these markers showed that only some of the postoperative scores predicted survival: high PLR had a negative prognostic impact, while high lymphocyte and PNI values had a positive effect on overall survival.

**Discussion:**

The circulating immune cells and their values integrated in the assessed prognostic scores suffer statistically significant changes after curative pancreatic surgery. Only the postoperative values of lymphocyte count, PLR, and PNI seem to influence the overall survival in PDAC.

**Trial registration:**

ClinicalTrials.gov–identifier NCT05025371.

## Introduction

Pancreatic ductal adenocarcinoma (PDAC) is the most frequent pancreatic tumor, comprising about 90% of all pancreatic neoplasms [1]. Pancreatic cancer is a highly aggressive malignancy with a survival rate of about 5% at 5 years [2]. The poor prognosis is also reflected by the incidence rate that is almost equal to the mortality rate [2]. Surgery is the only potentially curative treatment, integrated in a multimodal approach [3]; however, long-term results remain poor. Therefore, a better understanding of the biology of pancreatic cancer, as well as finding easy to use markers for diagnosis and prognosis would be useful in clinical practice [4].

Systemic inflammation has been recognized as a factor with an important role in the development of cancer [5, 6]. The systemic inflammatory response has been quantified through different scores (prognostic nutritional index, systemic immune-inflammation index) or ratios between different circulating immune cells (neutrophil to lymphocyte ratio, platelet to lymphocyte ratio, monocyte to lymphocyte ratio). Although these factors are gaining more interest even in pancreatic cancer, there is still controversy regarding their role in this type of cancer. Since these are easy to determine and easy to use scores, that might improve the management of PDAC, further investigations are necessary.

Our study intends to analyze the role of the available systemic immune-inflammatory scores on a cohort of patients with resectable PDAC from our surgical center. The objective is to assess the utility of these scores, calculated based on both preoperative and postoperative blood work, in predicting the prognosis of patients that underwent surgery with curative intent for PDAC. Another objective of our study is to assess the dynamic of these scores after curative surgery.

## Methods

Our study is a retrospective, single-center, observational cohort study. The study was approved by the Ethics Committee of Iuliu Hatieganu University of Medicine and Pharmacy (No. 112/15.04.2021), as well as the Ethics Committee of Regional Institute of Gastroenterology and Hepatology Prof. Dr. O. Fodor Cluj-Napoca (No. 4580/01.04.2021). The study was conducted based on a previously designed study protocol; the study was registered at ClinicalTrials.gov, having the identifier NCT05025371. The presented study will be reported according to STROCSS guidelines (strengthening the reporting of cohort studies in surgery) [7].

The study population consisted in the patients treated in the Surgical Department of Regional Institute of Gastroenterology and Hepatology Prof. Dr. O. Fodor Cluj-Napoca between January 2012 and December 2019. The last time of follow-up was April 2021.

We included only the patients with the final histopathological diagnosis of PDAC, who underwent pancreatic surgical resections. We excluded patients with other histopathological diagnoses, patients who underwent palliative interventions and patients with insufficient or incomplete data.

A number of 2396 records of patients with pancreatic tumors were identified in the selected period. After excluding the patients who were inoperable and who underwent palliative procedures, a number of 499 patients who underwent curative surgery was obtained. We then excluded the patients with other definitive histopathological diagnoses: 351 patients diagnosed with PDAC remained. An additional 39 patients were excluded due to insufficient data, so 312 patients were included in our study.

We gathered information from preoperative blood tests and postoperative blood tests. The preoperative blood tests used were those done at admission, before any treatment was administered. The postoperative blood work used was the one at discharge. All blood cell counts will be represented as count *10^3^/μL. Albumin was represented as g/dL. Based on these blood counts, we calculated different immune-inflammatory scores, using the following formulas:

- NLR was defined as neutrophil/lymphocyte count.
- LMR was defined as lymphocyte/monocyte count.
- PLR was defined as platelet/lymphocyte count.
- SII was defined as (platelet*neutrophil)/lymphocyte count.
- PNI was defined as 10*albumin + 5*lymphocyte count.

We analyzed the difference between the preoperative and postoperative values. Each parameter was correlated with the overall survival of patients. The overall survival (OS) has been defined as the time from the day of surgery to the time of death from any cause.

We have also gathered other histopathological parameters, like stage, grade of differentiation, nodal positivity (N), extranodal extension (ENE), perineural invasion (Pn), lymphatic invasion (L), and vascular invasion (V).

Our institute has a recommended follow-up protocol: check-ups at every 3 months in the first postoperative year, and afterwards, check-ups at every 6 months for the following 5 years.

Data analysis was performed using R 4.0.1. The cut-off point was chosen taking into consideration the Youden index. Normality of the distribution was calculated using Shapiro-Wilk test and histogram visualization and evaluating skewness and kurtosis. Non-normally distributed variables were represented as median (quartile 1, quartile 3). Difference in the preoperative and postoperative variables was performed using Mann-Whitney-Wilcoxon signed rank test. The correlation between an ordinal variable and a continuous variable was assessed using Kendall’s tau. Survival analysis was represented using Kaplan-Meier curves. Significance of the Kaplan-Meier curves was assessed using the log-rank test. Univariate survival analysis was performed using a univariate Cox proportional hazards model. A *p* value under 0.05 was considered statistically significant. Multivariable survival analysis was performed using a multivariable Cox proportional hazards model. In this model, we included the variables that were shown to be statistically significant in the univariable Cox proportional hazards model. The immunoinflammatory markers were not all included in the same model as there are certain components that are common in their calculation.

## Results

In the current study, we included a total of 312 patients with their general characteristics presented in Table 1.

**Table 1:** General characteristics of the cohort

Variable		*n* = 312
Age		63.4 ± 9.4^a^
Surgical intervention	PD	261 (83.65%)
	DP + Sp	27 (8.65%)
	DP	9 (2.9%)
	TP	9 (2.9%)
	TP + Sp	6 (1.9%)
Stage (AJCC 8^th^ edition)	IA	8 (2.56%)
	IB	33 (10.57%)
	IIA	53 (16.98%)
	IIB	181 (58.01%)
	III	37 (11.85%)
G	1	71 (22.75%)
	2	175 (56.08%)
	3	66 (21.15%)
R	0	199 (63.8%)
	1	113 (36.2%)
Pn	0	56 (17.94%)
	1	251 (80.44%)
L	0	91 (29.16%)
	1	221 (70.83%)
V	0	181 (58.01%)
	1	131 (41.98%)
N	0	93 (29.8%)
	1	182 (58.33%)
	2	37 (11.85%)
ENE	0	243 (77.88%)
	1	69 (22.11%)

*PD* pancreaticoduodenectomy, *DP* distal pancreatectomy, *Sp* splenectomy, *TP* total pancreatectomy, *G* grade of differentiation, *R* resection margin, *Pn* perineural invasion, *L* lymphatic invasion, *V* vascular invasion, *N* nodal involvement, *ENE* extranodal extension

^a^ Mean ± standard deviation

In this cohort, we observed that all the immune-inflammatory markers assessed presented significant changes after the surgery (Table 2).

**Table 2:** The values of immuno-inflammatory markers before and after the surgical intervention—value changes after surgery

Variable	Preoperative	Postoperative	*p* value
Neutrophils	5.10 (4.08, 6.25)	6.64 (5.04, 8.69)	< 0.0001
Lymphocytes	1.71 (1.32, 2.16)	1.50 (1.16, 2.00)	< 0.0001
Monocytes	0.47 (0.37, 0.61)	0.61 (0.46, 0.86)	< 0.0001
Platelets	272 (224, 327)	312 (233, 397)	< 0.0001
NLR	2.98 (2.22, 3.98)	4.32 (2.96, 6.32)	< 0.0001
LMR	3.73 (2.77, 4.77)	2.50 (1.85, 3.43)	< 0.0001
PLR	155 (125, 208)	200 (145, 285)	< 0.0001
SII	788 (563, 1122)	1336 (874, 2092)	< 0.0001
PNI	49 (45, 53)	38 (34, 42)	< 0.0001

Variables were represented as median (quartile 1, quartile 3)

Further, we assessed the impact on overall survival of the value of these markers before and after the surgical intervention. We chose to filter and dichotomize our data by comparing the values over percentile 75% with those under percentile 25%. This has been performed to determine if extreme values of the selected parameters have an impact regarding OS. Using this approach, we observed that only some of the postoperative values predicted survival. Patients with low postoperative lymphocyte count (less than 1.16 compared to greater than 2), high postoperative PLR (greater than 285 compared to less than 145), and low postoperative PNI (less than 34 compared to more than 43) had worse OS (Table 3). More specifically, high PLR had a negative prognostic impact, while high lymphocyte and PNI values had a positive effect on overall survival (Table 3).

**Table 3:** Univariate Cox proportional hazards model using the filtered and dichotomized immuno-inflammatory values on overall survival

Variable	Preoperative				Postoperative			
	OR	95% lower CI	95% upper CI	***p*** value	OR	95% lower CI	95% upper CI	***p*** value
Neutrophils	1.00	0.71	1.50	0.935	0.95	0.65	1.40	0.771
Lymphocytes	0.80	0.56	1.20	0.245	0.64	0.45	0.92	**0.016**
Monocytes	0.83	0.58	1.20	0.325	0.84	0.59	1.20	0.353
Platelets	0.87	0.60	1.30	0.450	0.96	0.67	1.40	0.841
NLR	1.20	0.90	1.80	0.243	1.20	0.82	1.70	0.373
LMR	1.00	0.71	1.50	0.886	0.88	0.61	1.30	0.506
PLR	1.10	0.78	1.70	0.492	1.90	1.30	2.70	**0.001**
SII	0.95	0.66	1.40	0.795	1.30	0.93	1.90	0.118
PNI	0.75	0.50	1.10	0.172	0.46	0.28	0.75	**0.002**

**Fig. 1:**
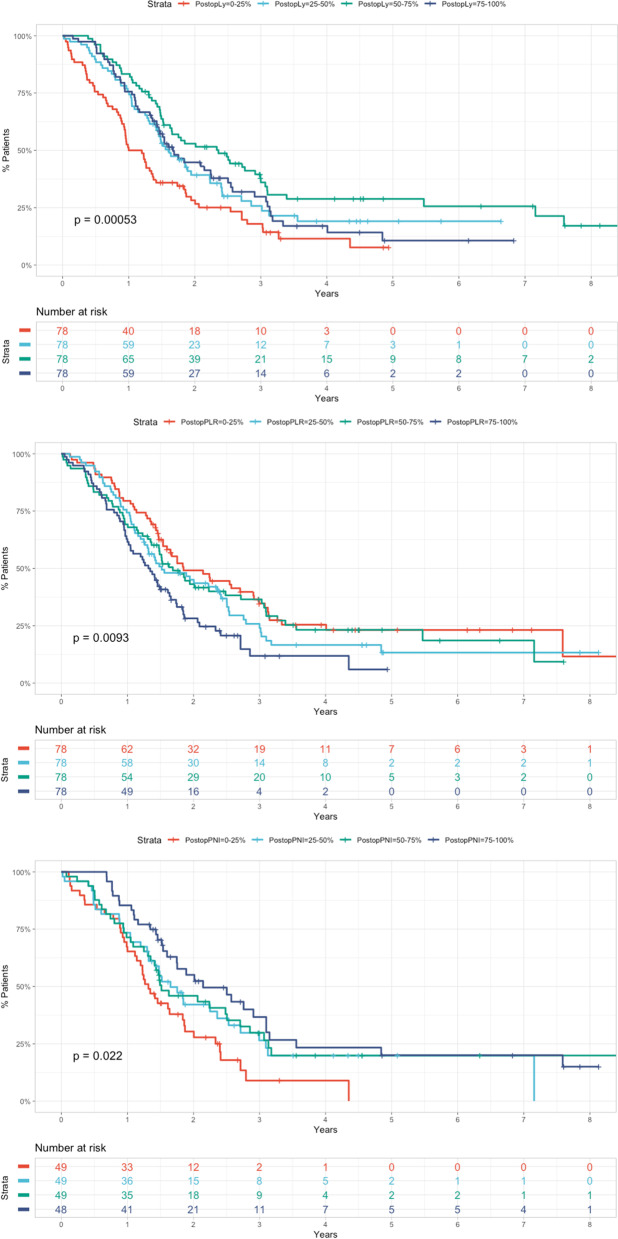
Kaplan-Meier curves showing the association of postoperative lymphocyte count, PLR, and PNI on overall survival

To better observe the effect of the significant immuno-inflammatory markers on overall survival, we plotted Kaplan-Meier curves for those considering all quartiles (Fig. 1).

Added to this, we also calculated the cut-off points for the aforementioned variables considering the death at 1, 2, and 3 years (Table 4). Given the relatively short overall survival of pancreatic adenocarcinoma, we also assessed what are the optimal cut-off values that have a relevance in predicting survival at 1, 2, and 3 years. As it can be observed, in the case of the three postoperative immunoinflammatory markers shown to be associated with overall survival (PLR, PNI, and lymphocytes), the cut-offs have similar values, showing the values around which, a cut-off can be clinically useful. It has to be mentioned that the use of a fixed cut-off, although may be attractive for the sake of simplicity, it does not necessarily reflect the reality, where multiple close cut-off values can have similar associations with overall survival.

**Table 4:** Cut-off points for death at 1, 2, and 3 years.

Variable	One year death cut-off	Two years death cut-off	Three years death cut-off
Preoperative neutrophils	4.94	4.94	7.00
Preoperative lymphocytes	2.05	2.09	1.86
Preoperative monocytes	0.28	0.33	0.33
Preoperative platelets	287.00	260.00	260.00
Preoperative NLR	2.58	4.61	1.94
Preoperative LMR	4.04	4.93	3.03
Preoperative PLR	122.16	187.34	187.34
Preoperative SII	819.78	803.40	803.40
Preoperative PNI	49.60	44.00	58.60
Postoperative neutrophils	6.13	6.07	4.82
Postoperative lymphocytes	1.19	1.53	1.44
Postoperative monocytes	0.60	0.34	0.64
Postoperative platelets	287.00	243.00	298.00
Postoperative NLR	3.34	3.56	3.56
Postoperative LMR	2.03	2.86	2.85
Postoperative PLR	211.27	218.26	252.63
Postoperative SII	2050.14	1178.49	1178.49
Postoperative PNI	42.65	42.70	40.25

We have assessed in a univariate Cox proportional hazards model the stage, grade, resection margin, lymphatic invasion, vascular invasion, node positivity, extranodal, postoperative PLR higher than 250, postoperative PNI higher than 40, and postoperative lymphocytes higher than 1.5 (Table 5). These cut-offs were chosen because of the previously presented results and rounded so they would be easier to be applied in the clinical setting.

**Table 5:** Univariate analysis on overall survival

Variable	HR	Lower 95% CI	Upper 95% CI	*p* value
Stage	1.20	1.00	1.50	**0.041**
R	1.00	0.78	1.30	0.93
Grade	1.40	1.20	1.70	**< 0.001**
Pn	0.93	0.67	1.30	0.656
L	1.70	1.20	2.20	**< 0.001**
V	1.40	1.10	1.80	**< 0.01**
N	1.60	1.30	2.00	**< 0.001**
ENE	1.60	1.20	2.10	**< 0.01**
Postoperative PLR > 250	1.50	1.20	2.00	**< 0.01**
Postoperative PNI > 40	0.62	0.44	0.88	**< 0.01**
Postoperative lymphocytes > 1.5	0.71	0.55	0.92	**0.01**

*G* grade of differentiation, *R* resection margin, *Pn* perineural invasion, *L* lymphatic invasion, *V* vascular invasion, *N* nodal involvement, *ENE* extranodal extension

We have also done a multivariate survival analysis, showing that the postoperative PNI, PLR, and lymphocytes have statistical significance in predicting the overall survival (Table 6).

**Table 6:** Multivariate analysis on overall survival

**Variable**	**HR**	**Lower 95% CI**	**Upper 95% CI**	***p*** **value**
Stage	1.00	0.78	1.40	0.853
G	1.4	1.19	1.80	**< 0.001**
L	1.00	0.67	1.60	0.913
V	1.10	0.81	1.50	0.579
N	1.40	1.00	2.00	**0.048**
ENE	1.20	0.85	1.70	0.309
Postoperative PLR > 250	1.60	1.20	2.10	**0.001**
**Variable**	**HR**	**Lower 95% CI**	**Upper 95% CI**	***p*** **value**
Stage	0.82	0.58	1.16	0.270
G	1.18	0.90	1.54	0.225
L	1.14	0.66	1.97	0.645
V	1.29	0.89	1.86	0.176
N	1.35	0.89	2.06	0.161
ENE	1.27	0.81	1.98	0.299
Postoperative PNI > 40	0.67	0.46	0.97	**0.035**
**Variable**	**HR**	**Lower 95% CI**	**Upper 95% CI**	***p*** **value**
Stage	1.00	0.75	1.31	0.977
G	1.46	1.19	1.79	**< 0.001**
L	1.07	0.70	1.63	0.764
V	1.09	0.82	1.46	0.540
N	1.42	1.01	1.98	**0.042**
ENE	1.20	0.86	1.68	0.285
Postoperative lymphocytes > 1.5	0.65	0.49	0.85	**< 0.01**

*G* grade of differentiation, *R* resection margin, *Pn* perineural invasion, *L* lymphatic invasion, *V* vascular invasion, *N* nodal involvement, *ENE* extranodal extension

## Discussion

Pancreatic cancer is a highly aggressive malignancy despite the current available treatments. Our study reunites a cohort of patients with PDAC, surgically treated in our tertiary center with experience in hepatobiliary surgery. We intended to analyze if the host’s immune response will influence the survival of patients; we quantified this immune response by known immune-inflammatory scores (NLR, PLR, LMR, PNI, and SII).

We retrospectively gathered a cohort of 312 patients, offering a large sample of patients compared to other published studies [8–10]. The patients included in the present study are classified as stages I through III; most of the included patients were stage IIB. The included patients did not receive any neoadjuvant treatment. Even tough other studies included all stages of PDAC [11] in their analysis, we believe that a more specific approach guided toward only resectable cases can offer important information for the clinical management of patients that undergo surgical treatment, minimizing the discrepancies caused by a heterogenous cohort of patients.

Alteration in the systemic inflammatory response and nutritional deterioration are encountered in cancer patients [12, 13], with the development and progression of cancer being closely related with the host-inflammatory response [14–16]. Several inflammation-based prognostic scores were developed for the evaluation of patients with cancer. Regarding the dynamic of the circulating immune cells in patients with cancer, it was observed that neutrophilia is an indicator for systemic inflammatory response and might play a role in carcinogenesis, lymphopenia reflects host immunosuppression and has a negative prognostic value, thrombocytosis is also a negative prognostic factor due to its possible role in cancer progression, while a high monocyte count is correlated with unfavorable prognosis [17]. These findings are the rationale behind the NLR, LMR, and PLR scores. The neutrophil-to-lymphocyte ratio, while initially developed for the evaluation of critically ill patients, has made its way into the evaluation of oncological patients [18–20], being widely used in various types of cancer. However, NLR and the other immune scores have not yet found their role in the clinical management of pancreatic cancer, controversies regarding their signification still exist [8]. The systemic immune-inflammation index was first described in hepatocellular carcinoma [21], as a non-invasive marker that uses an integrated index based on peripheral neutrophil, platelet, and lymphocyte counts; since then, its use has been extended to various types of cancer [22]. The prognostic nutritional index combines the evaluation of the immune and nutritional status, being first developed to evaluate postoperative complications in patients undergoing gastrointestinal surgery [23]; subsequently, more research has found that PNI has an important prognostic role in cancer patients [24].

Our study proved that important changes occur in the circulating immune cells populations after the resection of the tumor. The total neutrophil, monocyte, and platelet count, as well as the NLR, PLR, and SII significantly increased after surgery. On the other hand, the lymphocyte count, LMR, and PNI score decreased. Only a few other studies in medical literature explore the postoperative dynamic of these immune scores, most studies relying only on preoperative values [25]. The postoperative alterations might be caused by the surgical stress, as well as the removal of the tumor itself. Hoshimoto et al. [25] also reported significant postoperative changes in immune-inflammatory markers, except for PLR and LMR. Although, the reported dynamic was different that the one registered in our study—for example, the neutrophil count and the NLR decreased postoperatively.

In our analysis, no preoperative scores predicted overall survival, at any cut-off value used. Various studies in medical literature reported positive results regarding the prognostic power of various immune-inflammatory markers discussed in our study; negative results have been reported as well [9, 26–29]. Furthermore, a cut-off value for any of these variables was not established, different values being used in different studies [9, 26].

Interestingly, we did find prognostic value for three postoperative markers: the total lymphocyte count, PLR, and PNI score. A high PLR was associated with low survival, while a high lymphocyte count and PNI were associated with good survival. As mentioned before, there are very few reports regarding the importance of postoperative immune-inflammatory markers in the prognosis of PDAC. To our knowledge, this study is the first to report the importance of the early postoperative PLR and PNI as prognostic markers in PDAC. Kim et al. [30] analyzed the changes in the immune status after curative pancreatic resections, concluding that the total lymphocyte count and NLR at 1 and 6 months postoperatively are effective predictors for survival. The long-term dynamic of immune markers after pancreatic surgery in oncological patients is an unexplored subject that deserves further investigations.

The strength of the present study relies on the large sample size of patients, with homogenous characteristics: only pancreatic ductal adenocarcinoma in resectable stages, without neoadjuvant chemoradiotherapy. We are also one of the few studies that analyses the postoperative dynamic of immune-inflammatory factors. Limitations are related especially to the retrospective nature of the study. We only analyzed the impact on the overall survival, the retrospective design did not permit the correlation of the immune-inflammatory scores with disease specific complications.

Since the results are promising, but not specific enough to be replicated on a large scale and used in clinical practice, we believe that future research should further explore the topic of immune-inflammatory markers in PDAC with a focus on specific subtypes of immune cells and their role in the progression of cancer. Even more so, since the dynamic of all the immune-inflammatory markers seems to be modified postoperatively, we believe that studying these markers at longer intervals after surgery can offer in depth information regarding their long-term dynamic.

The circulating immune cells as well as their values integrated in prognostic scores (NLR, LMR, PLR, PNI, SII) suffer statistically significant changes after curative pancreatic surgery. The preoperative immune-inflammatory scores analyzed in the present article are not predictive for the overall survival of patients. Only the postoperative values of lymphocyte count, PLR, and PNI seem to influence the overall survival in PDAC. A high PLR had a negative prognostic impact, while high lymphocyte and PNI values had a positive effect on overall survival.

## Data Availability

The datasets used and/or analyzed during the current study are available from the corresponding author on reasonable request.

## Abbreviations

PDAC: Pancreatic ductal adenocarcinoma
PNI: Prognostic nutritional index
SII: Systemic immune-inflammation index
NLR: Neutrophil to lymphocyte ratio
PLR: Platelet to lymphocyte ratio
MLR: Monocyte to lymphocyte ratio

## References

[1] Haeberle L, Esposito I (2019). Pathology of pancreatic cancer. Transl Gastroenterol Hepatol.

[2] Ducreux M, Cuhna AS, Caramella C, Hollebecque A, Burtin P, Goéré D (2015). Cancer of the pancreas: ESMO Clinical Practice Guidelines for diagnosis, treatment and follow-up. Ann Oncol.

[3] Lambert A, Schwarz L, Borbath I, Henry A, van Laethem J-L, Malka D (2019). An update on treatment options for pancreatic adenocarcinoma. Ther Adv Med Oncol.

[4] Harris M, Brekke M, Dinant G-J, Esteva M, Hoffman R, Marzo-Castillejo M (2020). Primary care practitioners’ diagnostic action when the patient may have cancer: an exploratory vignette study in 20 European countries. BMJ Open.

[5] Proctor MJ, Morrison DS, Talwar D, Balmer SM, Fletcher CD, O’Reilly DS (2011). A comparison of inflammation-based prognostic scores in patients with cancer. A Glasgow Inflammation Outcome Study. Eur J Cancer.

[6] Colotta F, Allavena P, Sica A, Garlanda C, Mantovani A (2009). Cancer-related inflammation, the seventh hallmark of cancer: links to genetic instability. Carcinogenesis..

[7] Agha R, Abdall-Razak A, Crossley E, Dowlut N, Iosifidis C, Mathew G (2019). STROCSS 2019 Guideline: strengthening the reporting of cohort studies in surgery. Int J Surg.

[8] Ahmad J, Grimes N, Farid S, Morris-Stiff G (2014). Inflammatory response related scoring systems in assessing the prognosis of patients with pancreatic ductal adenocarcinoma: a systematic review. Hepatobiliary Pancreat Dis Int.

[9] Mowbray NG, Griffith D, Hammoda M, Shingler G, Kambal A, Al-Sarireh B (2018). A meta-analysis of the utility of the neutrophil-to-lymphocyte ratio in predicting survival after pancreatic cancer resection. HPB..

[10] Oh D, Pyo J-S, Son BK (2018). Prognostic roles of inflammatory markers in pancreatic cancer: comparison between the neutrophil-to-lymphocyte ratio and platelet-to-lymphocyte ratio. Gastroenterol Res Pract.

[11] Lee SH, Chung MJ, Kim B, Lee HS, Lee HJ, Heo JY (2017). The significance of the prognostic nutritional index for all stages of pancreatic cancer. Nutr Cancer.

[12] McMillan DC (2009). Systemic inflammation, nutritional status and survival in patients with cancer. Curr Opin Clin Nutr Metab Care.

[13] Arrieta O, Michel Ortega RM, Villanueva-Rodríguez G, Serna-Thomé MG, Flores-Estrada D, Diaz-Romero C (2010). Association of nutritional status and serum albumin levels with development of toxicity in patients with advanced non-small cell lung cancer treated with paclitaxel-cisplatin chemotherapy: a prospective study. BMC Cancer.

[14] Argilés JM, Busquets S, López-Soriano FJ (2003). Cytokines in the pathogenesis of cancer cachexia. Curr Opin Clin Nutr Metab Care.

[15] Mantovani A, Allavena P, Sica A, Balkwill F (2008). Cancer-related inflammation. Nature..

[16] Kinoshita A, Onoda H, Imai N, Iwaku A, Oishi M, Fushiya N (2012). Comparison of the prognostic value of inflammation-based prognostic scores in patients with hepatocellular carcinoma. Br J Cancer.

[17] Krakowska M, Dębska-Szmich S, Czyżykowski R, Zadrożna-Nowak A, Potemski P (2018). The prognostic impact of neutrophil-to-lymphocyte ratio, lymphocyte-to-monocyte ratio, and platelet-to-lymphocyte ratio in patients with advanced colorectal cancer treated with first-line chemotherapy. Prz Gastroenterol.

[18] Walsh SR, Cook EJ, Goulder F, Justin TA, Keeling NJ (2005). Neutrophil-lymphocyte ratio as a prognostic factor in colorectal cancer. J Surg Oncol.

[19] Zahorec R (2001). Ratio of neutrophil to lymphocyte counts--rapid and simple parameter of systemic inflammation and stress in critically ill. Bratisl Lek Listy.

[20] Zhang Y, Wu W, Dong L, Yang C, Fan P, Wu H (2016). Neutrophil to lymphocyte ratio predicts persistent organ failure and in-hospital mortality in an Asian Chinese population of acute pancreatitis. Medicine (Baltimore).

[21] Hu B, Yang X-R, Xu Y, Sun Y-F, Sun C, Guo W (2014). Systemic immune-inflammation index predicts prognosis of patients after curative resection for hepatocellular carcinoma. Clin Cancer Res.

[22] Chen J-H, Zhai E-T, Yuan Y-J, Wu K-M, Xu J-B, Peng J-J (2017). Systemic immune-inflammation index for predicting prognosis of colorectal cancer. World J Gastroenterol.

[23] Onodera T, Goseki N, Kosaki G (1984). Prognostic nutritional index in gastrointestinal surgery of malnourished cancer patients. Nihon Geka Gakkai zasshi.

[24] Hua X, Long Z-Q, Huang X, Deng J-P, He Z-Y, Guo L (2020). The value of prognostic nutritional Index (PNI) in predicting survival and guiding radiotherapy of patients with T1-2N1 breast cancer. Front Oncol.

[25] Hoshimoto S, Hishinuma S, Shirakawa H, Tomikawa M, Ozawa I, Ogata Y (2020). Validation and clinical usefulness of pre- and postoperative systemic inflammatory parameters as prognostic markers in patients with potentially resectable pancreatic cancer. Pancreatology..

[26] Zhou Y, Cheng S, Fathy AH, Qian H, Zhao Y (2018). Prognostic value of platelet-to-lymphocyte ratio in pancreatic cancer: a comprehensive meta-analysis of 17 cohort studies. Onco Targets Ther.

[27] Hu R, Ma J, Hu G (2018). Lymphocyte-to-monocyte ratio in pancreatic cancer: prognostic significance and meta-analysis. Clin Chim Acta.

[28] Li S, Tian G, Chen Z, Zhuang Y, Li G (2019). Prognostic role of the prognostic nutritional index in pancreatic cancer: a meta-analysis. Nutr Cancer.

[29] Jomrich G, Gruber ES, Winkler D, Hollenstein M, Gnant M, Sahora K (2020). Systemic immune-inflammation index (SII) predicts poor survival in pancreatic cancer patients undergoing resection. J Gastrointest Surg.

[30] Kim EY, Hong TH (2019). Changes in total lymphocyte count and neutrophil-to-lymphocyte ratio after curative pancreatectomy in patients with pancreas adenocarcinoma and their prognostic role. J Surg Oncol.

